# Gallbladder adenomyomatosis: Diagnosis and management

**DOI:** 10.1016/j.ijscr.2021.106089

**Published:** 2021-06-11

**Authors:** Atef Mejri, Khaoula Arfaoui, Ahmed Omri, Jasser Rchidi, Mohamed Ali Mseddi, Sarra Saad

**Affiliations:** a Department of General Surgery, Jendouba Hospital, Tunisia; b Anaesthesiology and Reanimation Department, Jendouba Hospital, Tunisia; c Tunis El Manar University, Tunisia

**Keywords:** Gallbladder, Adenomyomatosis, Adenocarcinoma, Cholecystectomy, Surgery, Case report

## Abstract

**Introduction:**

Gallbladder adenomyomatosis is a benign acquired gallbladder disease. It can mimic cancer on radiological findings, leading to a diagnostic dilemma. The management and prognosis of these two gallbladder anomalies are entirely different. Therefore, it is essential to recognize the pathognomonic features of gallbladder adenomyomatosis is in order to accurately diagnose this pathology. This paper presents two encountered cases of gallbladder adenomyomatosis is, their diagnostic modalities as well as the algorithmic approach of their management. These two-case reports have been reported in line with the SCARE Criteria 2020 [1].

**Presentation of case:**

Patient-1 was symptomatic. He was explored by an abdominal ultrasound picturing gallbladder wall thickening while the biopsy showed pleomorphic proliferation of inflammatory cells. An examination of the liver with MRI was indicated, showing diffuse parietal thickening with multiple cystic pockets. He underwent laparoscopic cholecystectomy with simple operative follow-up. Patient 2 was asymptomatic, a staging CT scan of acute pancreatitis revealed focal mural thinking of the gallbladder wall. A liver MRI showed an intramural cystic formation in the vesicular fundus. Given the inconclusive imaging results, laparoscopic cholecystectomy was performed. Histological examination confirmed the diagnosis of GA.

**Discussion:**

Adenomyomatosis is usually asymptomatic. Imaging can confirm the diagnosis of gallbladder adenomyomatosis without the need for invasive procedures such as vesicular biopsy. Histologic examination can also confirm the diagnosis when cholecystectomy is done. High resolution ultra-sound is the most efficient radiological examination. Laparoscopic cholecystectomy is the gold standard for symptomatic GA or radiological suspicion of a gallbladder cancer.

**Conclusion:**

The practitioner should always consider gallbladder carcinoma before confirming the GA, as they share the same features but with a far worse prognosis. The likelihood of malignancy depends on radiological characteristics. In the case of inconclusive findings, cholecystectomy is justified.

## Highlights

• Gallbladder adenomyomatosis is a **frequent** but **underestimated** pathology as it is usually asymptomatic.
• The diagnosis is most commonly made **post-operatively** on histological examination of a cholecystectomy specimen.
• Gallbladder adenomyomatosis may mimic **cancerous lesions** on radiological findings, which leads to a **diagnostic dilemma**.
• **Clinical symptomatology** as well as the **reliability of the radiological diagnosis** guide therapeutic management.

## 1 Introduction

Gallbladder adenomyomatosis (GA) is a benign acquired gallbladder disease [2], with a generally favorable prognosis [3,4]. It affects both sexes equally [4] and has a peak incidence in the sixth and seventh decades of life [2]. Gallbladder adenomyomatosis is the dominant cause of benign gallbladder lesions, accounting for 40% of them. However, it may mimic cancerous lesion on radiological findings, which leads to a diagnostic dilemma since the management and outcome of these two pathologies are entirely different. Hence, the need to recognize pathognomonic features of gallbladder adenomyomatosis in order to characterize its nature and provide proper treatment. The localized type is described as the most common in elderly, whereas, in children the diffuse type was the most encountered type [5]. This article reviews two cases of gallbladder adenomyomatosis. The aim of this case presentation is outlining the clinical features of gallbladder adenomyomatosis, determining the most effective radiological investigation, identifying the pathognomonic radiological findings, and differentiating it from gallbladder neoplastic pathologies as well as to describe the most efficient therapeutic approach. These two-case reports have been reported in line with the SCARE Criteria 2020 [1].

## 2 Presentation of cases

### 2.1 Patient 1

**Fig. 1:**
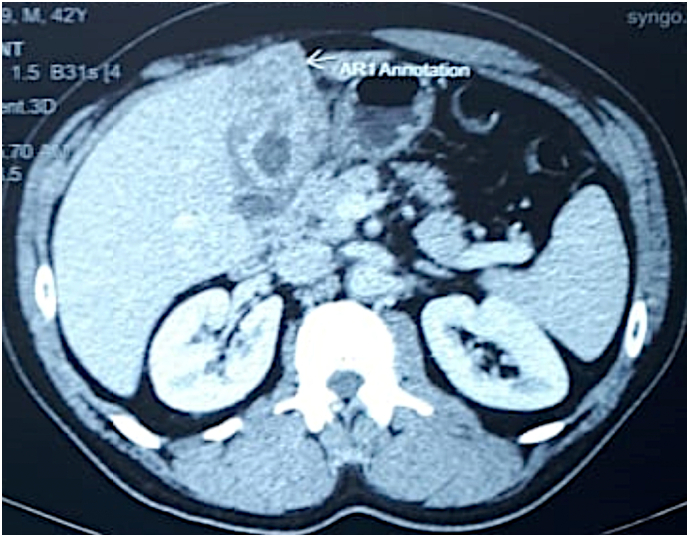
CT axial slice showing diffuse parietal thickening of the gallbladder with invasion of segment V and antropyloric region of the stomach.

**Fig. 2:**
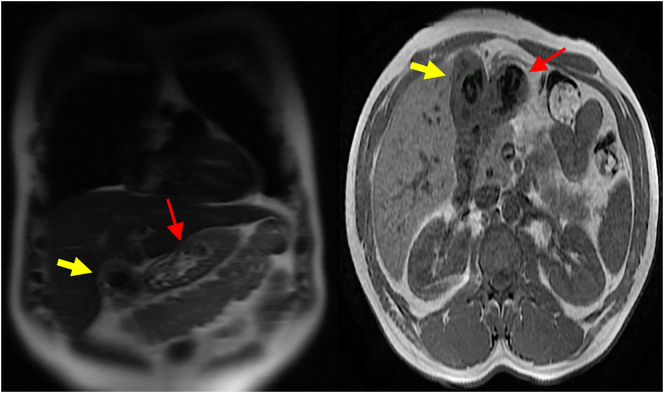
MRI view showing diffuse parietal thickening of the gallbladder (yellow arrow) with multiple cystic pockets and invasion of the antropyloric region of the stomach (red arrow).

**Fig. 3:**
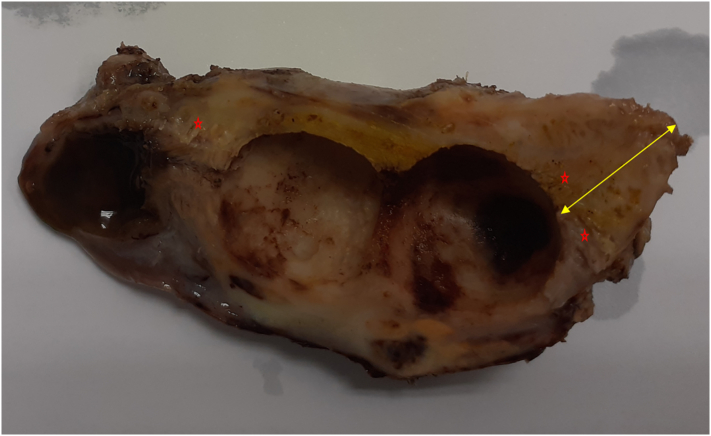
Trimmed gross specimen showing enlargement of gallbladder wall (double arrow) with numerous small cysts (star).

**Fig. 4:**
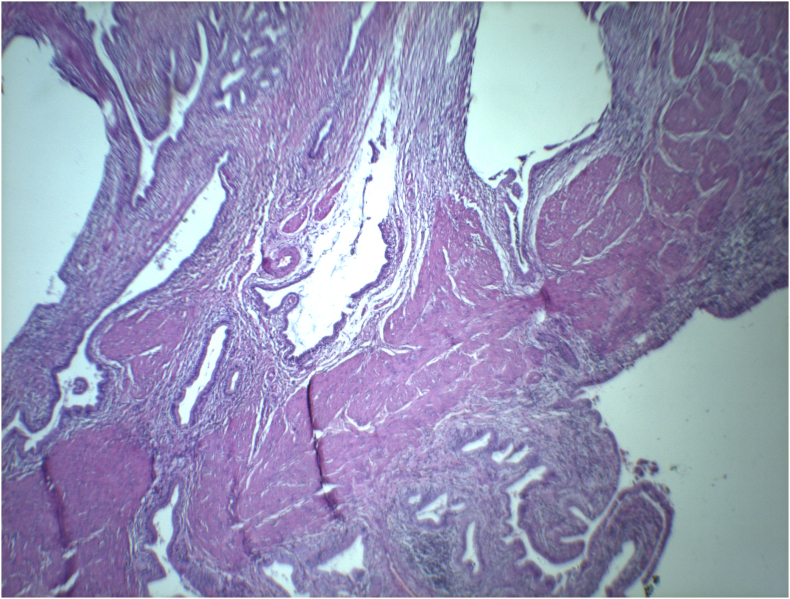
The corresponding microscopic description shows cystically dilated glands invaginating into the thickened muscular wall. Hematoxylin and eosin; magnification 100×.

A 42-year-old male patient, presented to our surgery out-patient clinic with an abdominal discomfort evolving for 4 months with no associated fever or jaundice. The patient did not have any changes in his bowel habit. He has no past medical history expect type 2 diabetes treated with Metformin and Glimepiride, with no family history. He had not undergone any surgical procedure. He denied any tobacco or illicit drug use. His physical examination was unremarkable, with no abdominal tenderness or palpable mass. Routine laboratory investigation showed no significant findings. Abdominal ultrasonography showed irregular thickening of gallbladder wall with images of reverberation artifacts. Due to the unavailability of an MRI, an abdominal CT-scan was performed, to clarify the nature of the encountered lesion. It revealed circumferential heterogeneous irregular parietal thickening of gallbladder rmeasuring19 mm causing extrinsic compression of segment V of the liver and the antropyloric region of the stomach (Fig. 1). No upper gastrointestinal endoscopy was performed since the extrinsic nature of the compression of the antropyloric region was evident on the CT scan. An ultrasound-guided fine-needle aspiration biopsy was performed twice to exclude malignant process. The first one was inconclusive. The second anatomopathological examination showed a proliferation of spindle cells on an inflammatory background with rather mono-formed nuclei and arranged in short bundles suggesting in the first place an inflammatory myofibroblastic tumor with absence of carcinomatous proliferation on these biopsies. The patient underwent an abdominal MRI showing diffuse parietal thickening measuring 1 cm with multiple cystic pockets (Fig. 2). At the end of this investigation, the most likely diagnosis was gallbladder adenomyomatosis. The patient underwent laparoscopic cholecystectomy with no intraprocedural complications. He was discharged on the third post-operative day. The final pathological finding revealed the following: Gross examination showed an expansion of gallbladder wall with numerous small cysts (Fig. 3), and histology showed multiple Rokitansky-Aschoff sinuses prolapsing between muscle bundles (Fig. 4). The post-operative course was uneventful. The diagnosis of gallbladder adenomyomatosis was therefore established.

### 2.2 Patient 2

A 62-year-old female was admitted to our surgery department for a hypertriglyceridemia-induced acute pancreatitis with no associated organ failure. She had uncontrolled dyslipidemiaandgrade1 hypertension with no previous abdominal surgery with no family history. Severity-grading abdominal CT scan was performed on day three of its symptomatology and revealed a turgescent pancreas with inflammatory changes in the peripancreatic fat. It also revealed fortuitously a focal mural thinking of the gallbladder wall. Complementary MRI was warranted to better characterize the described lesion and it showed an intramural cystic formation within the vesicular fundus. Given the unsolved diagnosis with inconclusive imaging data, a laparoscopic cholecystectomy was done15 days after admission. Gross examination of the gallbladder found focal fundibular thickened gallbladder wall with epithelial invaginations within the underlying muscular layer giving the appearance of epithelial cystic pockets. Histology examination corroborated the suspected diagnosis of GA.

## 3 Discussion

The pathogenesis of GA remains incompletely understood and controversial [3]: an association between gallbladder stones, being a necessary precursor, and chronic inflammatory changes has been highlighted in many studies [2]. It has been suggested that epithelial growth is stimulated by constant inflammation.

GA is usually asymptomatic [6,7] and incidentally detected when undergoing routine ultrasound exam [8,9]. However, it may cause right sided upper quadrant sporadic pain similar to biliary colic, which could be attributed to the commonly coexisting gallbladder stones [7,9,10]. In fact, 60% of these cholecystectomy specimens are lithiasic [2,11]. Moreover, it is generally in patients who are operated on for symptomatic vesicular lithiasis that adenomyomatosis is discovered on histological examination of the specimen. No episodes of jaundice or change in color of urine or stools were noted [12]. The clinical presentation may also be misleading and only resumed in a lingering pain as described by Teelucksingh S. et al. [4,7]. Patient 1 has also consulted several general practitioners for a puzzling abdominal discomfort and has been put on various symptomatic treatments like analgesics, anti-spasmodic, anti-inflammatory medicines without improvement in a period of 4 months. The patient underwent two gallbladder-biopsies to clarify the diagnosis. Despite the fact that physical exam is unremarkable in most subjects, although tenderness was reported by Michal Pasierbek et al. [5]. Hematologic and biochemical test results in patients with GA are usually normal [9].

The abdominal ultrasound can accurately diagnose gallbladder adenomyomatosis [3,13]: mural thickening (diffuse, focal, annular), intra-parietal cystic images with low echogenicity, intramural calculi with “comet-tail” artifacts pictured by distal sonographic shadowing emanating from the small echogenic intramural foci [11], which is highly specific for gallbladder adenomyomatosis [14]. Depending on the location and extent [13] of GB wall thickening, GA is classified into three [14] morphological types: focal, segmental or diffuse [9]: The focal type is the most common pattern and usually involves the gallbladder fundus [15]. Rarely, it involves the whole organ [2]. Gallbladder wall thickening must be regarded with caution to spot the arguments in favor of malignancy: asymmetrical focal stenosing thickening, or thickening greater than 10 mm, adjacent liver invasion and presence of locoregional lymph nodes or metastases. The CT appearance of GA is similar to the sonographic findings. This radiological exam is limited because it is unable to demonstrate the small cystic spaces and concretions that are sonographically apparent due to reverberation artifact. Thus, the US appearance is more accurate than the CT findings for the diagnosis of GA [11,16]. Abdominal MRI may be useful for further characterization in case of diagnostic doubt [4,13]. In fact, MRI has a higher sensitivity (73% vs 80,3) and specificity (96,3% vs 98,2) than US as demonstrated in Bang et al. search comparing these three radiological tools [16]. High resolution US has comparable results to MRI [16]. However, considering its availability and affordability, high resolution US represents the imaging modality of choice for the diagnosis of GA [3]. The constellation of diffuse gallbladder wall thickening with intramural diverticula forms the “rosary sign” and “pearl necklace sign” distinguishing this diagnosis from other etiologies [14].

Due to a specificity rate of imaging findings close to 100%, anatomopathological arguments can be omitted in the presence of pathognomonic radiological signs cited above. The pathognomonic histologic features of gallbladder adenomyomatosis include large, deep and dendritic Rokitansky-Aschoff sinuses accompanied by wall muscle thickening [17]. No histological justification for these pathological processes was found. With no intrinsic malignant potential [3,13,18] and no extra hepatic spread in spite of its proliferative features [19], GA is not considered a premalignant lesion [13].

**Fig. 5:**
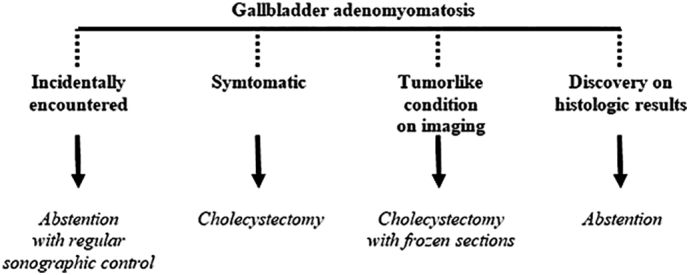
Algorithm approach for the management of gallbladder adenomyomatosis.

There is no universally agreed-upon guideline for the management of GA at present. The questions are how reliable is the diagnosis of GA on imaging and how high is the chance of malignant change for a genuine GA [9]. In the cases where the correct diagnosis is made pre operatively via radiological tools, GA management depends on its clinical presentation: If adenomyomatosis is found incidentally and is asymptomatic, the patient is not offered further treatment, in order to minimize unnecessary surgery [4], and a wait-and-see approach can be conducted. However, the radiological diagnosis must be beyond any doubt about the possibility of GB cancer. If the radiological results are inconclusive, a cholecystectomy is justified [2]. This approach requires careful follow-up and after multiple imaging procedures to spot signs of thickening or irregularity, which could be indications for cholecystectomy [8]. Routine ultrasounds are the choice of imaging for these patients with an interval of 3–6 months [9]. To our knowledge, there is no survey evaluating the duration of this morphological surveillance. Symptomatic GA is an indication for cholecystectomy which results in complete relief of symptoms. [2]. In case of inconclusive imaging findings: when it is difficult to distinguish adenomyomatosis from carcinomas on radiological features, cholecystectomy is warranted [4]. When opened longitudinally, GA can be suspected grossly when the cut surface have segmental, localized, or diffuse thickening of the wall, honeycombed appearance containing numerous multifaceted intraluminal calculi [11,20]. Intra-operative frozen section should be offered to exclude gallbladder carcinoma (GC). Extended surgery should be planned in advance if GC is confirmed intra-operatively [9]. The postoperative discovery of GA in a cholecystectomy specimen does not require any special surveillance [2]. Our study allowed us to propose a decisional algorithm illustrated by the (Fig. 5). To our knowledge, no study has been conducted on the preferred surgical approach. Consequently, since it has no local invasion trait, surgery was undergone through laparoscopy. A more thorough study with a larger number of patients could be useful to elucidate the outcome of cholecystectomy in GA, and to decide the best surgical approach.

## 4 Conclusion

The practitioner should always consider gallbladder carcinoma before confirming the GA, as they share the same features but with a far worse prognosis. The likelihood of malignancy depends on radiological characteristics. In case of inconclusive findings, cholecystectomy is justified. Symptomatic GA requires cholecystectomy. If GA is discovered on gallbladder specimen, no further requirement is needed. If GA is incidentally discovered, ultrasound monitoring is necessary.

## Abbreviations

GA: gallbladder adenomyomatosis
CT: computerized tomography
MRI: Magnetic resonance imaging
US: ultrasound
GC: gallbladder carcinoma

## Ethical approval

Ethical approval has been exempted by our institution because this is a case report and no new studies or new techniques were carried out.

## Funding sources

All the authors declare that this research didn't receive any specific grant from funding agencies in the public, commercial, or not-for-profit sectors.

## CRediT authorship contribution statement

Atef MEJRI: Operated on the patient, drafting and revising the manuscript, literature research.

Khaoula ARFAOUI: Drafting the manuscript and literature research.

Mohamed Ali MSEDDI: Drafting the manuscript and literature research.

Sarra SAAD: Drafting the manuscript and literature research.

Ahmed OMRI: Drafting the manuscript and literature research.

Jasser RCHIDI: Drafting the manuscript and literature research.

## Guarantor

The guarantor for this case report is Atef MEJRI.

Registration of research studies

Not applicable.

## Consent

Written informed consent was obtained from the patient for publication of this case report and accompanying images. A copy of the written consent is available for the Editor-in-Chief of this journal on request.

## Provenance and peer review

Not commissioned, externally peer-reviewed.

## Declaration of competing interest

All the authors certify that there is no conflict of interest regarding the material discussed in the manuscript.
